# Correlative mass spectrometry imaging, applying time‐of‐flight secondary ion mass spectrometry and atmospheric pressure matrix‐assisted laser desorption/ionization to a single tissue section

**DOI:** 10.1002/rcm.8022

**Published:** 2017-12-19

**Authors:** N. Desbenoit, A. Walch, B. Spengler, A. Brunelle, A. Römpp

**Affiliations:** ^1^ Chair of Bioanalytical Sciences and Food Analysis University of Bayreuth Bayreuth Germany; ^2^ Institute of Inorganic and Analytical Chemistry, Justus Liebig University Giessen Germany; ^3^ Research Unit Analytical Pathology Helmholtz Zentrum München Deutsches Forschungszentrum für Gesundheit und Umwelt (GmbH) Germany; ^4^ Institut de Chimie des Substances Naturelles, CNRS UPR2301, Université Paris‐Sud, Université Paris‐Saclay Avenue de la Terrasse Gif‐sur‐Yvette France

## Abstract

**Rationale:**

Mass spectrometry imaging (MSI) is a powerful tool for mapping the surface of a sample. Time‐of‐flight secondary ion mass spectrometry (TOF‐SIMS) and atmospheric pressure matrix‐assisted laser desorption/ionization (AP‐MALDI) offer complementary capabilities. Here, we present a workflow to apply both techniques to a single tissue section and combine the resulting data for the example of human colon cancer tissue.

**Methods:**

Following cryo‐sectioning, images were acquired using the high spatial resolution (1 μm pixel size) provided by TOF‐SIMS. The same section was then coated with a para‐nitroaniline matrix and images were acquired using AP‐MALDI coupled to an Orbitrap mass spectrometer, offering high mass resolution, high mass accuracy and tandem mass spectrometry (MS/MS) capabilities. Datasets provided by both mass spectrometers were converted into the open and vendor‐independent imzML file format and processed with the open‐source software MSiReader.

**Results:**

The TOF‐SIMS and AP‐MALDI mass spectra show strong signals of fatty acids, cholesterol, phosphatidylcholine and sphingomyelin. We showed a high correlation between the fatty acid ions detected with TOF‐SIMS in negative ion mode and the phosphatidylcholine ions detected with AP‐MALDI in positive ion mode using a similar setting for visualization. Histological staining on the same section allowed the identification of the anatomical structures and their correlation with the ion images.

**Conclusions:**

This multimodal approach using two MSI platforms shows an excellent complementarity for the localization and identification of lipids. The spatial resolution of both systems is at or close to cellular dimensions, and thus spatial correlation can only be obtained if the same tissue section is analyzed sequentially. Data processing based on imzML allows a real correlation of the imaging datasets provided by these two technologies and opens the way for a more complete molecular view of the anatomical structures of biological tissues.

## 1 INTRODUCTION

Mass spectrometry imaging (MSI) is the major and very active analytical method among the various techniques aiming to map the surface of a sample, capable of providing simultaneously the spatial distribution of a wide range of molecules directly from biological samples in a single run.^1^ Secondary ion mass spectrometry (SIMS) and matrix‐assisted laser desorption/ionization (MALDI) are the two main techniques commonly used for MSI. Briefly, MSI is based on a focused primary ion beam (SIMS) or laser beam (MALDI) which irradiates spot after spot over a delimited sample surface. The desorbed ions of the region scanned are transferred and separated according to their *m*/*z* values in the mass analyzer. These two techniques allow access to the distribution of several classes of biomolecules from the surface of a tissue section.^2^, ^3^, ^4^, ^5^

Time‐of‐flight (TOF)‐SIMS involves the bombardment of a sample by a focused beam of mono‐ or polyatomic ions, which induces desorption/ionization of secondary ions from the surface of the sample.^6^, ^7^, ^8^ It also offers the possibility of localizing various ions produced from molecules, mainly lipids, drugs, xenobiotics and metabolites, with *m*/*z* up to 1000–1500, good mass resolution (*M*/Δ*M* = 8000 (full width at half maximum) at *m*/*z* > 500) and a high lateral resolution from 400 nm to 1–2 μm. This makes TOF‐SIMS a method of choice for the micrometric‐scale analysis of lipids or other kinds of small molecules in biological samples.^5^ Moreover, no matrix coating is required, i.e. no surface modification is made. One of the main important breakthroughs in TOF‐SIMS during the last fifteen years concerns polyatomic ion sources. The introduction of such polyatomic ion sources and, in particular, ion guns providing metal clusters (e.g. bismuth and gold clusters) has improved the desorption/ionization of intact ions from molecules, significantly expanding the application of TOF‐SIMS from a mapping tool of elements or small mass fragments to a powerful molecular microscope used in various fields, ranging from materials characterization to biological tissue imaging.^6^, ^8^, ^9^, ^10^ Despite this improvement, two main limitations still exist: the high fragmentation rate induced by the high collision energy from the primary ion beam and the lack of tandem mass spectrometry capabilities.^11^, ^12^, ^13^ After the first attempts a few years ago,^14^, ^15^ the latter issue is going to be addressed in the near future, with the recent advent of SIMS instruments with TOF/TOF and/or high‐resolution Orbitrap mass analyzers.^16^, ^17^

MALDI imaging was described initially by Spengler et al^18^, ^19^ and tissue imaging was first shown by Caprioli et al.^20^ Until recently, the main limitation of the MALDI method for MSI was its spatial resolution, which was typically in the range 50–200 μm. The Spengler group developed an efficient atmospheric pressure scanning microprobe MALDI method with a focused laser beam providing a high spatial resolution of 1.4 μm.^21^ Moreover, coupling this with an orbital trapping mass spectrometer offers high mass resolution, mass accuracy and MS/MS capabilities.^21^ In addition, an atmospheric pressure (AP)‐MALDI ion source is perfectly suited for investigating biological samples and allows the detection of a wide range of biomolecule classes, including metabolites,^22^, ^23^ lipids^24^ and peptides/proteins.^25^

MSI is now widely used in many applications, mainly in biological sciences and medical research,^26^, ^27^, ^28^, ^29^ but also in cultural heritage research.^30^ Correlated imaging has become an emerging strategy to combine complementary information from different analytical techniques.^31^ The Cooks group combined desorption electrospray ionization (DESI) and MALDI imaging using a single tissue section,^32^ achieving lipid and protein imaging by DESI‐MS and MALDI‐MS, respectively.^32^ Despite the improvement concerning polyatomic ion sources, Brunelle et al showed the need to combine molecular information from TOF‐SIMS and MALDI‐MS imaging, and the possibility of performing a MALDI imaging experiment on the same sample after TOF‐SIMS imaging.^33^ Eijkel et al combined MALDI and SIMS imaging datasets applied to human cerebellum tissue.^34^ Touboul et al also combined these two imaging techniques to study skin and kidney biopsies of patients suffering from Fabry disease by mapping globotriaosylceramides and digalactosylceramides,^35^ showing good complementarity between the two techniques based on the identification and localization of biomolecules. In addition, Chughtai et al combined the elemental and small‐molecule distribution provided by high lateral resolution SIMS with the specific distribution of lipids and peptides/proteins provided by MALDI for the study of musculoskeletal tissue.^36^

Imaging dataset processing is a great challenge. The main difficulty for biologists or clinicians is to analyze, merge, compare and correlate data provided by different instruments on the same platform. Moreover, MSI data comprise a complex and huge dataset containing all relevant properties correlated to the mass spectral data. Vendors of MS instruments and many bio‐informatics groups have come up with several pieces of software to analyze MSI datasets. Consequently, a common data format known as imzML^37^ has been developed over the past few years.^38^ The vendor‐neutral data format imzML facilitates the flexible sharing of MSI data and their visualization into various software tools available without restriction to a proprietary vendor.^38^ Additional details are provided in Roempp et al.^39^ One of the most relevant examples is the data processing of a multicenter study.^40^ The authors analyzed adjacent sections of mouse brain in five laboratories situated mainly in Europe and the USA. Five different instruments were used: MALDI‐TOF/TOF, Orbitrap, QTOF, FT‐ICR and TOF‐SIMS. The imaging dataset was converted into imzML format using the appropriate converter tools^37^ and displayed in a common open‐source software to facilitate exchange and the comparison.^40^

In the study reported here, we defined a workflow based on the investigation of lipids combining TOF‐SIMS and AP‐MALDI‐Orbitrap. In addition, this multimodal approach using these two imaging methods offers a strong complementarity, due, on the one hand, to the precise localization of biomolecules by the high spatial resolution provided by TOF‐SIMS, and, on the other, to the identification/confirmation of molecular structures by the high mass accuracy, high mass resolution, high lateral resolution and MS/MS capability of the AP‐MALDI‐MS setup. Imaging data were converted into the standard imzML format and MS images generated using an open‐source software. The workflow was applied to only one tissue section of human colon tumor to correlate information.

## 2 EXPERIMENTAL

### 2.1 Chemicals for MALDI imaging

Water and acetone (HPLC grade), trifluoroacetic acid and *para*‐nitroaniline (pNA) were purchased from Fluka (Neu Ulm, Germany).

### 2.2 Tissue samples

Serial cryo‐sections of human colon cancer (thickness: 12 μm) were cut at −20 °C using a CM1950‐S cryostat (Leica, Wetzlar, Germany) and deposited on glass slides coated with indium tin oxide. The samples were dried in vacuum under a pressure of a few hectopascals for 15 min before the SIMS analyses. Optical images were recorded with a BX51 microscope (Olympus, Rungis, France) equipped with ×1.25 to ×50 lenses and a Color View I camera, monitored by Cell^B^ software (Soft Imaging Systems GmbH, Münster, Germany).

### 2.3 TOF‐SIMS imaging

The experiments were performed using a commercial TOF‐SIMS IV mass spectrometer (ION‐TOF GmbH, Münster, Germany). This mass spectrometer, described in detail elsewhere,^8^ is fitted with a bismuth liquid metal ion gun delivering Bi_*n*_^*q*+^ bismuth cluster ions (Bi_3_^+^ ions were selected). A low‐energy electron flood gun was activated between two primary ions pulses to neutralize the sample surface, causing only minimum damage.^41^

Only one mode of operation of the primary ion column was used during the experiments, which is called a “high‐current bunched mode”,^34^, ^42^ ensuring both a beam focus of 2 μm and a pulse duration of less than 1 ns, thus enabling an excellent mass resolution, *M*/Δ*M* = 5 × 10^3^ (full width at half maximum), at *m*/*z* 500. The Bi_3_^+^ primary ion current, measured at 10 kHz with a Faraday cup on a grounded sample holder, was *ca* 0.40 pA in this mode. Both positive and negative ion modes of image acquisition were used. Images of the human colon with a field of view of 500 μm × 500 μm containing 512 × 512 pixels were recorded, leading to a pixel size of 1 μm. Consequently, in this mode the pixel stepsize was smaller than the beam diameter (2 μm), leading to oversampling. Another mode of operation could be used, which combines a higher spatial resolution of *ca* 400 nm and a mass resolution of *M*/Δ*M* = 8 × 10^3^, thanks to a delayed extraction of the secondary ions.^43^ However in the present case the “high‐current bunched mode” was preferred because it ensures the fastest acquisition time. Under these conditions, the fluence (also called the primary ion dose density) was maintained at 5.0 × 10^11^ ions cm^−2^, which is below the so‐called static SIMS limit.^44^ Because of the very low initial kinetic energy distribution of the secondary ions, the relationship between the TOF and the square root of the *m*/*z* value is always linear over the whole mass range. The calibration was always internal and the signals used for the initial calibration were those of H^+^, H_2_^+^, H_3_^+^, C^+^, CH^+^, CH_2_^+^, CH_3_^+^ and C_2_H_5_^+^ ions in positive ion mode and H^−^, C^−^, CH^−^, CH_2_^−^, C_2_^−^, C_3_^−^ and C_4_H^−^ in negative ion mode. The mass calibration could eventually be refined by adding well‐identified ions of higher mass, such as fatty acid carboxylates and deprotonated vitamin E, to further improve mass accuracy.^45^, ^46^ The data acquisition software used was SurfaceLab 6.2 (ION‐TOF GmbH).

### 2.4 AP‐MALDI‐MS imaging

After the static SIMS imaging experiments, a uniform matrix layer (pNA, 10 mg mL^−1^ in 1:1 acetone/water, 0.1% trifluoroacetic acid) was applied to the section using a pneumatic sprayer.^47^ The MALDI‐MS imaging analyses were performed using a high lateral resolution atmospheric pressure imaging ion source (AP‐MALDI10, TransMIT GmbH, Giessen, Germany) coupled to an orbital trapping mass spectrometer (Q Exactive, Thermo Fisher Scientific GmbH, Bremen, Germany).^21^ The mass spectrometer was operated in positive ion mode at a mass resolution of 140,000 at *m*/*z* 200 over a mass range of *m*/*z* 700 to 900. The ion source was equipped with a nitrogen laser (*λ* = 337 nm), operating at a repetition rate of 60 Hz, for desorption/ionization. A useful spatial resolution from biological tissue down to a pixel size of 5 μm has been reported using this ion source.^2^, ^24^ Internal mass calibration was performed using a lipid ion signal as a lock mass [PC(34:1) + K]^+^ ion at *m*/*z* 798.54096 in positive ion mode, resulting in a mass accuracy better than 2 ppm. Positive lipid ion fragmentation was performed to identify and confirm some molecular structures of lipids by high‐energy collisional dissociation. The isolation window for the precursor was set to ±0.5 u. The mass resolution for MS/MS was set to *R* = 70,000 (at *m*/*z* 200).

### 2.5 Data processing

Image datasets from TOF‐SIMS (.ITM files from ION‐TOF were exported into. GRD by SurfaceLab software) and AP‐MALDI‐MS (.RAW file from Thermo Fisher Scientific) were converted into imzML using the “toImzmlModule” converter developed by Commissariat à l'Energie Atomique et aux Energies Alternatives (CEA, Saclay, France), and the “RAW to imzML” converter developed by Justus Liebig University, respectively.^38^ As a result, the imzML files were processed using MSiReader, a free open‐source MSI software. This vendor‐neutral interface was built on Matlab by Robichaud et al.^48^ The ion selection bin width (*m*/*z* window) of the images generated from the MALDI‐MS dataset was Δ*m*/*z* = 0.01, and Δ*m*/*z* = 0.2 for the TOF‐SIMS dataset. Additional details of the imzML conversion and processing are available online.^37^ Note that the TOF‐SIMS and AP‐MALDI‐MS images have not been normalized or interpolated.

### 2.6 Histological staining

The section imaged was stained after MSI measurement to compare the histological features. Using the same section, hematoxylin and eosin (H&E) staining was performed after removing the pNA matrix with 100% ethanol.

## 3 RESULTS AND DISCUSSION

**Figure 1:**
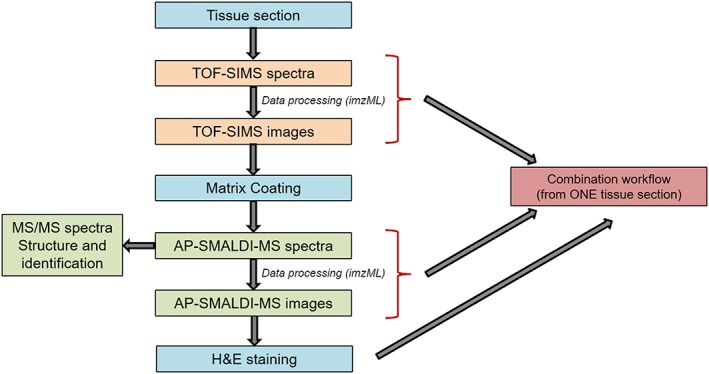
Workflow for correlative mass spectrometry imaging [Color figure can be viewed at wileyonlinelibrary.com]

The workflow presented for correlating MSI combining TOF‐SIMS and AP‐MALDI‐MS on a single biological section involves several steps and is presented in Figure 1. The first step to consider in multimodal imaging experiments is the sample support (critical parameter). Several types of plates can be used for MSI. The most commonly used in TOF‐SIMS is the silicon wafer, because it offers many essential qualities, such as perfect flatness, conductivity and inexpensiveness.^49^ For AP‐MALDI, standard microscope glass slides were used because they offer the possibility of carrying out histological staining on the same tissue section. Consequently, the choice of support on which to deposit the section was a glass slide coated with indium tin oxide. The latter offers a good compromise between the conductivity properties (needed for SIMS) to perform the workflow using a single tissue section with the two MSI methods, and the transparency to stain using a standard tissue fixation technique. After tissue cryo‐sectioning and deposition onto the coated slide, a direct analysis of the sample of interest was achieved using the high lateral resolution (about 1 μm) provided by TOF‐SIMS. This imaging technique requires no pretreatment of the tissue section and does not damage the sample. After SIMS analysis, a uniform matrix layer was applied to the tissue section and AP‐MALDI‐MS imaging experiments were conducted with high mass accuracy (<2 ppm with an internal calibration) and mass resolution (*R* = 140,000 at *m/z* 200). The MS/MS capability of AP‐MALDI‐MS was then used directly on the tissue to identify and confirm the molecular structures. Furthermore, H&E staining was carried out to correlate the distribution of the ions with the histoanatomical features. Finally, the imaging datasets obtained using the two techniques were converted into imzML^38^ and displayed using MSiReader,^48^ an open‐source software tool, to compare and tentatively correlate the ion images and histological staining. This workflow was applied to a human colon cancer tissue section. Colon cancer is among the most commonly diagnosed cancers in Europe and is a frequent cause of mortality, the second after lung cancer. Aging, heredity and inflammatory chronic disease are the main reasons for this pathology.

**Figure 2:**
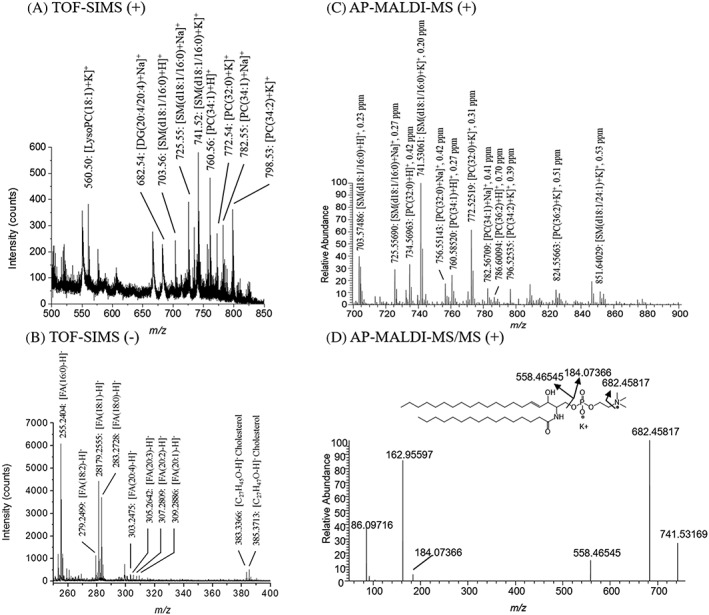
TOF‐SIMS mass spectra in positive ion mode (A) and negative ion mode (B). AP‐MALDI‐MS mass spectrum in positive ion mode in a mass range of *m*/*z* 700–900 (C) and AP‐MALDI‐MS/MS product ion spectrum of *m*/*z* 741.53061 assigned to [SM(34:1) + K]^+^ with the characteristic product ions also shown (D). The lipids identified were assigned based on accurate mass. RMS values were calculated for AP‐MALDI acquisition

Representative mass spectra for each mode are shown in Figure 2. The mass spectra were acquired in positive and negative ion modes with TOF‐SIMS (Figures 2A and 2B) and in positive ion mode only with AP‐MALDI‐MS (Figure 2C) in the infiltrated submucosa. The phosphatidylcholine head group, cholesterol and vitamin E were detected in positive ion mode (Figure S1, supporting information). Lysophosphatidylcholine (Lyso‐PC), phosphatidylcholine (PC) and sphingomyelin (SM) were also detected (Figure 2A). The negative secondary ion mass spectrum was dominated by deprotonated fatty acids (FAs) and cholesterol (Figure 2B).

The choices of matrix and coating method are the key steps of MALDI imaging. The Yappert group proved that the pNA matrix was an advantageous alternative to the use of 2,5‐dihydroxybenzoic acid.^50^, ^51^, ^52^ The promising pNA matrix improved significantly the sensitivity of the classes of phospholipids, such as PCs and SMs, in comparison with the 2,5‐dihydroxybenzoic acid matrix.^50^, ^51^, ^52^ Furthermore, the Bunch group compared α‐cyano‐4‐hydroxycinnamic acid and pNA matrices, showing the competitive effect of pNA in comparison with α‐cyano‐4‐hydroxycinnamic acid in terms of lipid ion intensities using MALDI imaging with an intermediate pressure ion source.^53^ After pNA matrix deposition, the AP‐MALDI‐MS experiment was conducted for a mass range of *m*/*z* 700–900 in positive ion mode (Figure 2C). PC and SM ions dominated the mass spectrum. The molecular structures were based on the high mass accuracy, and the root mean square (RMS) was calculated for each of them. The RMS values allow the evaluation of the variation for the *m*/*z* values given during the whole measurement; they were better than 1 ppm and confirmed the high mass accuracy and the stability of the instrument during the measurement. One of the main concerns was the sample quality after the TOF‐SIMS analysis, as the sample placed in the main chamber of the TOF‐SIMS was subjected to an ultrahigh vacuum (1 × 10^−9^ mbar) during the acquisition. The tissue sample was transported from the Institut de Chimie des Substances Naturelles in Gif‐sur‐Yvette (vicinity of Paris, France) to the University of Giessen (60 km north of Frankfurt airport, Germany). Nevertheless, the tissue remained intact and no specific damage could be observed. Matrix application for high‐resolution MALDI imaging is a challenging experimental procedure. It is therefore remarkable that high‐quality AP‐MALDI data could be acquired after the section had been kept under vacuum in the TOF‐SIMS chamber for several hours. Fresh tissue sections are generally not freeze‐dried and contain a significant amount of water to work in optimal conditions in our high‐resolution AP‐MALDI system. Consequently, the two MSI desorption techniques, TOF‐SIMS and AP‐MALDI‐MS, allowed the detection of similar phospholipid ions, such as PC and SM, in positive ion mode. On the other hand, AP‐MALDI‐MS allowed the enhancement of the detection and identification of phospholipids based on the high mass resolution and accuracy.

The FA distributions provided by TOF‐SIMS could be correlated to the signal of the corresponding phospholipids supplied in positive ion mode by MALDI‐MS. Figure 2D shows the product ion spectrum (MS/MS) of *m*/*z* 741.53064 recorded from tissue with the AP‐MALDI‐QExactive setup, confirming the assignment made according to the accurate mass for this sphingomyelin [SM(34:1) + K]^+^. The high‐energy collisional dissociation fragmentation of this lipid ion species (energy fixed at 25 arbitrary units) showed the expected neutral losses of a part of the choline residue N(CH_3_)_3_ (59 u), corresponding to the product ion detected at *m*/*z* 682.45817, of the PC head group C_5_H_15_NPO_4_ (183 u), corresponding to the product ion at *m*/*z* 558.46545, and the detection of the PC head group at *m*/*z* 184.07368. It also included the peak for the phosphonoacetaldehyde cationized by potassium at *m*/*z* 162.95594, which confirms potassium as an adduct, thus facilitating identification. Consequently, this MS/MS experiment partially confirmed the molecular structure of the sphingomyelin. The most abundant species of PCs and SMs detected by AP‐MALDI imaging and identified by MS/MS measurements are indicated in Table S1 (supporting information).

**Figure 3:**
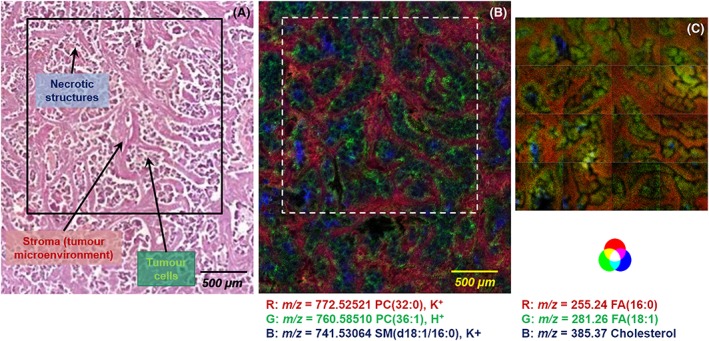
Human colon cancer tissue section. Optical image of the infiltrated submucosa corresponding to the region imaged by AP‐MALDI‐MS. The black square corresponds to the region imaged by TOF‐SIMS (A). AP‐MALDI‐MS image RGB overlay in positive ion mode: [PC(32:0) + K]^+^ at *m*/*z* 772.52521 ± 0.31 ppm (red), [PC(36:1) + H]^+^ at *m*/*z* 760.58510 ± 0.27 ppm (green) and [SM(d18:1/16:0) + K]^+^ at *m*/*z* 741.51061 ± 0.20 ppm (blue) (B). Overlay of adjacent juxtaposition of 16 TOF‐SIMS images in negative ion mode in the RGB system: carboxylates [FA(16:0) − H]^−^ at *m*/*z* 255.24 (red) and [FA(18:1) − H]^−^ at *m*/*z* 281.26 (green), and cholesterol [C_27_H_47_O − H]^−^ at *m*/*z* 385.37 (blue) (C) [Color figure can be viewed at wileyonlinelibrary.com]

Colon cancer spreads through the mucosa layer to the submucosa layer. Cancer cells infiltrate the submucosa and modify the cellular and extracellular composition.^53^ Figure 3A shows an optical image of the submucosa infiltrated. The necrotic structures, tumor microenvironment (desmoplastic tumor stroma) and tumor cells were assigned after H&E staining and were labeled accordingly. The displayed area was mapped by AP‐MALDI corresponding to a field of view of 2550 μm × 3000 μm, with a step size of 10 μm (255 × 300 pixels). The black square indicates the region that was scanned by TOF‐SIMS. This second area corresponds to the combination/juxtaposition of 16 adjacent images of 500 μm × 500 μm each, with a pixel size fixed at 1 μm (512 × 512 pixels). The imzML conversion allows access to a large choice of software tools and the corresponding key features.^37^ In this study we used the open‐source software MSiReader,^48^ which offers the opportunity to overlay ion images with an optical picture, such as histological staining. Moreover, the main advantage of using a unique software is the possibility of using the same settings for visualization, such as the color panel/interpolation settings, leading to a better combination and correlation/comparison of the imaging dataset. Figure 3B shows an overlay of several mass signals provided by AP‐MALDI‐MS: *m*/*z* 772.52521 (red), *m*/*z* 760.58510 (green) and *m*/*z* 741.51093 (blue), which were assigned to the potassium adduct of PC(32:0), protonated PC(36:1) and the potassium adduct of SM(34:1), respectively. The two PCs, PC(32:0) and PC(36:1), were localized in the tumor microenvironment and the tumor cells, respectively. The sphingomyelin was accumulated mainly in the necrotic areas. Figure 3C shows the three‐color overlay between three TOF‐SIMS negative ion images: *m*/*z* 255.24 (red), *m*/*z* 281.26 (green) and *m*/*z* 385.37 (blue), corresponding to carboxylates FA(16:0), FA(18:1) and deprotonated cholesterol, respectively. The latter was accumulated mainly in the necrotic areas, while the two FAs, FA(16:0) and FA(18:1), were localized in the tumor microenvironment and the tumor cells, respectively.

**Figure 4:**
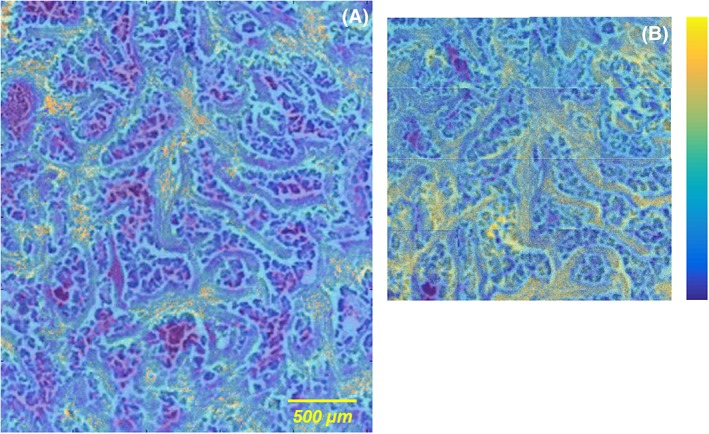
Co‐registration of hematoxylin and eosin staining with the AP‐MALDI ion image of the [PC(32:0) + K]^+^ (A) and the TOF‐SIMS ion image of the [FA(16:0) − H]^−^ (B) using the “parula” colormap [Color figure can be viewed at wileyonlinelibrary.com]

The co‐registration feature of an optical image with ion images provided by MSiReader was used for AP‐MALDI and TOF‐SIMS, to correlate and confirm the distribution of the ions in the histoanatomical substructures (Figure 4). For this purpose, identical settings for generating images were used and the “parula” colormap was chosen. After alignment of the H&E‐stained image to match with the MS image, the transparency was adjusted, confirming the distribution of the PC [PC(32:0) + K]^+^ as detected by AP‐MALDI‐MS in the tumor microenvironment (Figure 4A). In the same way, each of the 16 individual adjacent images generated by TOF‐SIMS were aligned with the optical view, confirming the main distribution of the palmitic acid [FA(16:0) − H]^−^ in the tumor microenvironment (Figure 4B).

The TOF‐SIMS and AP‐MALDI data can be compared and correlated using the flexibility of the imzML format. One of the main benefits of data processing using this common format is the possibility of displaying MS images with identical settings for visualization (color scale, normalization and interpolation). Consequently, TOF‐SIMS and AP‐MALDI showed a high correlation between the distribution of the FAs detected in negative ion mode and the PCs detected in positive ion mode in the tumor microenvironment. Moreover, cholesterol and sphingomyelin ions seemed to be co‐localized in the necrotic areas. Formation of these areas could be the consequence of the apoptosis mechanism. The Setou group showed the distribution of sphingomyelin in colon cancer liver metastasis tissue by MALDI‐MS imaging.^54^

The workflow presented using only one tissue section has shown, for the first time, a high image quality, due to the very high spatial resolution provided by TOF‐SIMS (*ca* 1 μm) and the very competitive spatial resolution provided by the efficient AP‐MALDI (10 μm). The reproducibility of the workflow is demonstrated using another kind of tissue section. Mouse brain was used for this purpose and showed a high correlation. The mass spectra showed a similar lipid profile in negative ion mode using the two MSI techniques (Figure S2, supporting information). In both cases, mass spectra were dominated by the sulfatides (STs). A common lipid ion at *m*/*z* 888 tentatively assigned to the sulfatide [ST(42:2) − H]^–^ was chosen and used to generate the TOF‐SIMS and AP‐MALDI images, showing the same distribution in the hippocampus area (Figure S3, supporting information). This demonstrates that the described workflow results in a high reproducibility and can be applied to other tissue types.

## 4 CONCLUSIONS

The workflow applied to a single human colon cancer sample, combining the most commonly used mass spectrometry imaging technologies TOF‐SIMS and AP‐MALDI‐Orbitrap, showed high spatial correlation and complementary molecular information. Improvements in the MALDI imaging spatial resolution allowed a much better spatial correlation with TOF‐SIMS. This requires the sequential analysis of a single tissue section, in contrast to the parallel investigation of adjacent tissue sections as carried out in previous investigations combining MALDI‐TOF and TOF‐SIMS. This fact has to be considered in the sample preparation procedure concerning, for example, sample support, sample handling and histological staining.

Data processing is a critical step in such a multimodal approach. We have therefore used the open data format imzML and open‐source software. The main benefits of the imzML format, such as flexibility in data analysis and access to a large choice of software tools, allowed a direct comparison and correlation of the different MS imaging methods.

The emerging technology of multimodal imaging significantly expands the capabilities for revealing the molecular complexity in tissue of both healthy and diseased state. Consequently, this approach could be used to obtain a more compete and detailed understanding of pathological changes on a molecular level, for example, by combining fast SIMS measurements of highest spatial resolution with high molecular information MALDI measurements.

## Supporting information


**Figure S1:** TOF‐SIMS mass spectra in positive ion mode in the mass range of *m/z* 150 – 250 (A), and *m/z* 300 – 500 (B).
**Table S1:** Product ions detected in MS^2^ spectra from SMALDI‐MS in positive ion mode.
**Figure S2:** TOF‐SIMS mass spectrum in negative ion mode (top) and AP‐MALDI‐MS mass spectrum in negative ion mode in mass range of *m/z* 700 – 950 (bottom). The lipids identified are assigned with the experimental mass measured and root mean square (RMS) values were calculated for the AP‐MALDI acquisition.
**Figure S3:** Mouse brain: Optical image of the hippocampus area corresponding to the region mapped by AP‐MALDI‐MS, and the black square corresponds to the region imaged by the TOF‐SIMS (A); AP‐MALDI‐MS image in negative ion mode in the “gray” colormap: [ST(42:2) ‐ H]^‐^ at *m/z* 888.62512 (B); Adjacent juxtaposition of ten TOF‐SIMS images in negative mode in the “gray” colormap: [ST(42:2) ‐ H]^‐^ at *m/z* 888.59 (C).
