# Supplementary material for: Correlative mass spectrometry imaging, applying time‐of‐flight secondary ion mass spectrometry and atmospheric pressure matrix‐assisted laser desorption/ionization to a single tissue section
Source: Rapid Commun Mass Spectrom. 2017 Dec 19;32(2):159–66. doi: 10.1002/rcm.8022 (PMC5838509; doi:10.1002/rcm.8022)

**Supporting information**

The TOF-SIMS measurements have been performed using the “high-current bunched mode,” and the Bi_3_^+^ primary ion current, measured at 10 kHz with a Faraday cup on the grounded sample holder, is ~0.40 pA in this mode. A low-energy electron flood gun is activated between two primary ions pulses to neutralize the sample surface with the minimum damage. The TOF-SIMS images of mouse brain with a field of view of 250 x 250 µm were recorded without stage movement just by rastering the primary ion beam randomly. In this case, the number of pixels was 256 × 256, leading to a pixel size of 1 µm. The pixel size is chosen as half of the primary ion beam size, resulting in a slight oversampling. Under these conditions, the fluence (also called the primary ion dose density) was maintained at 1.0 × 10^12^ ions/cm^-2^, which is below the so-called static SIMS limit. Both positive and negative ion modes of image acquisition were used. The relationship between the TOF and the square root of *m/z* is always linear over the whole mass range because of the very low initial kinetic energy distribution of the secondary ions. The calibration was always internal and signals used for initial calibration were those of H^+^, H_2_^+^, H_3_^+^, C^+^, CH^+^, CH_2_^+^, CH_3_^+^, C_2_H_5_^+^ ions, and H^-^, C^-^, CH^-^, CH_2_^-^, C_2_^-^, C_3_^-^, C_4_H^-^ in positive and negative ion mode, respectively. The data acquisition software used was SurfaceLab 6.2 (ION-TOF GmbH, Münster, Germany).

After the static SIMS imaging experiments, a uniform matrix layer (pNA, 10 mg/mL in 1:1 acetone/water, 0.1 % TFA) was applied to the section using a home-built pneumatic sprayer. Another advantage not mentioned previously is the possibility of using a pNA matrix in a negative ion mode. The MALDI MS imaging analyses were performed using a high lateral resolution atmospheric pressure imaging ion source (AP-MALDI10, TransMIT GmbH, Giessen, Germany) coupled to an orbital trapping mass spectrometer (QExactive, Thermo Fisher Scientific GmbH, Bremen, Germany). The mass spectrometer was operated in negative ion mode at a mass resolution of 140,000 at *m/z* 200 over a mass range of *m/z* 700 – 950. The ion source was equipped with a nitrogen laser (λ = 337 nm), operating at a repetition rate of 60 Hz, for desorption/ionization. A usable spatial resolution from biological tissue has been reported down to 5 µm pixel size with this ion source. Internal mass calibration was performed using the isotope form of lipid ion signals as a lock mass [PI(38:4) - H]^-^ ion at *m/z* 886.55479 in negative ion mode, resulting in a mass accuracy better than 2 ppm.

**Figure S1:** TOF-SIMS mass spectra in positive ion mode in the mass range of *m/z* 150 – 250 (A), and *m/z* 300 – 500 (B).

**
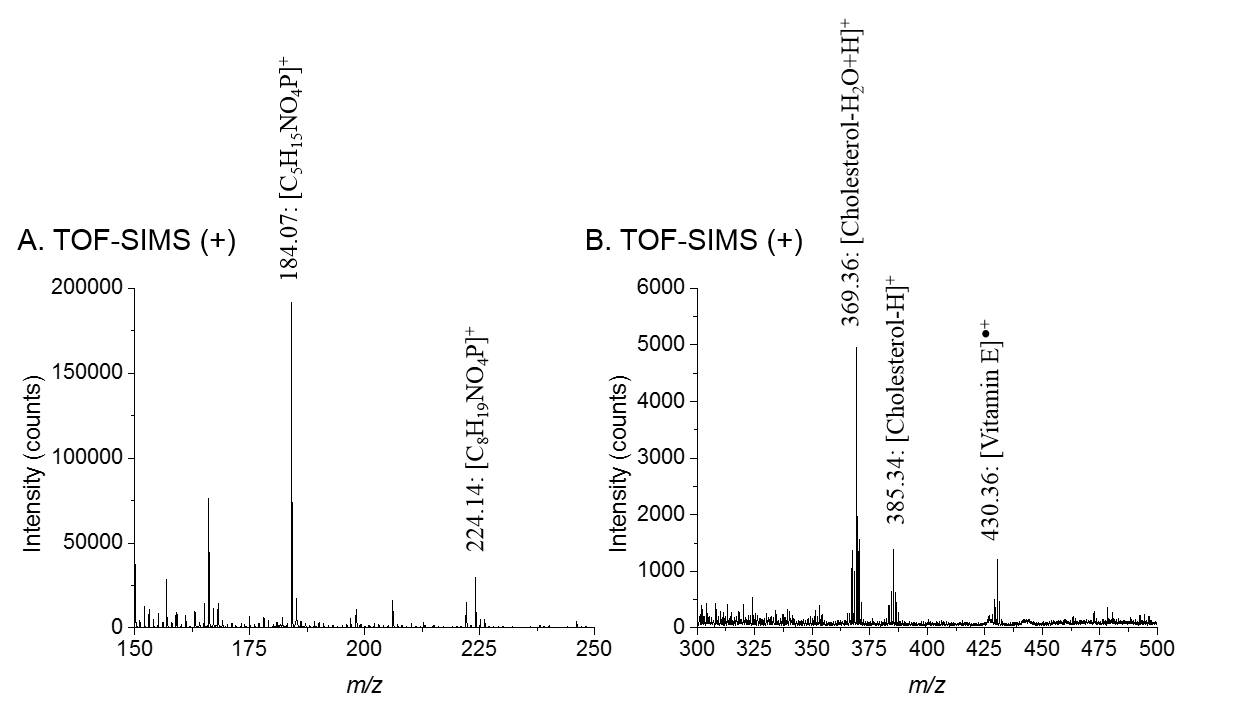
**

**Table S1:** Product ions detected in MS² spectra from SMALDI-MS in positive ion mode.


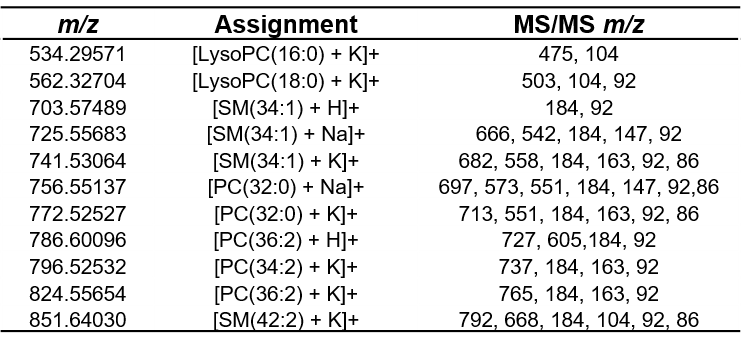


**Figure S2:** TOF-SIMS mass spectrum in negative ion mode (top) and AP-MALDI-MS mass spectrum in negative ion mode in mass range of *m/z* 700 – 950 (bottom). The lipids identified are assigned with the experimental mass measured and root mean square (RMS) values were calculated for the AP-MALDI acquisition.


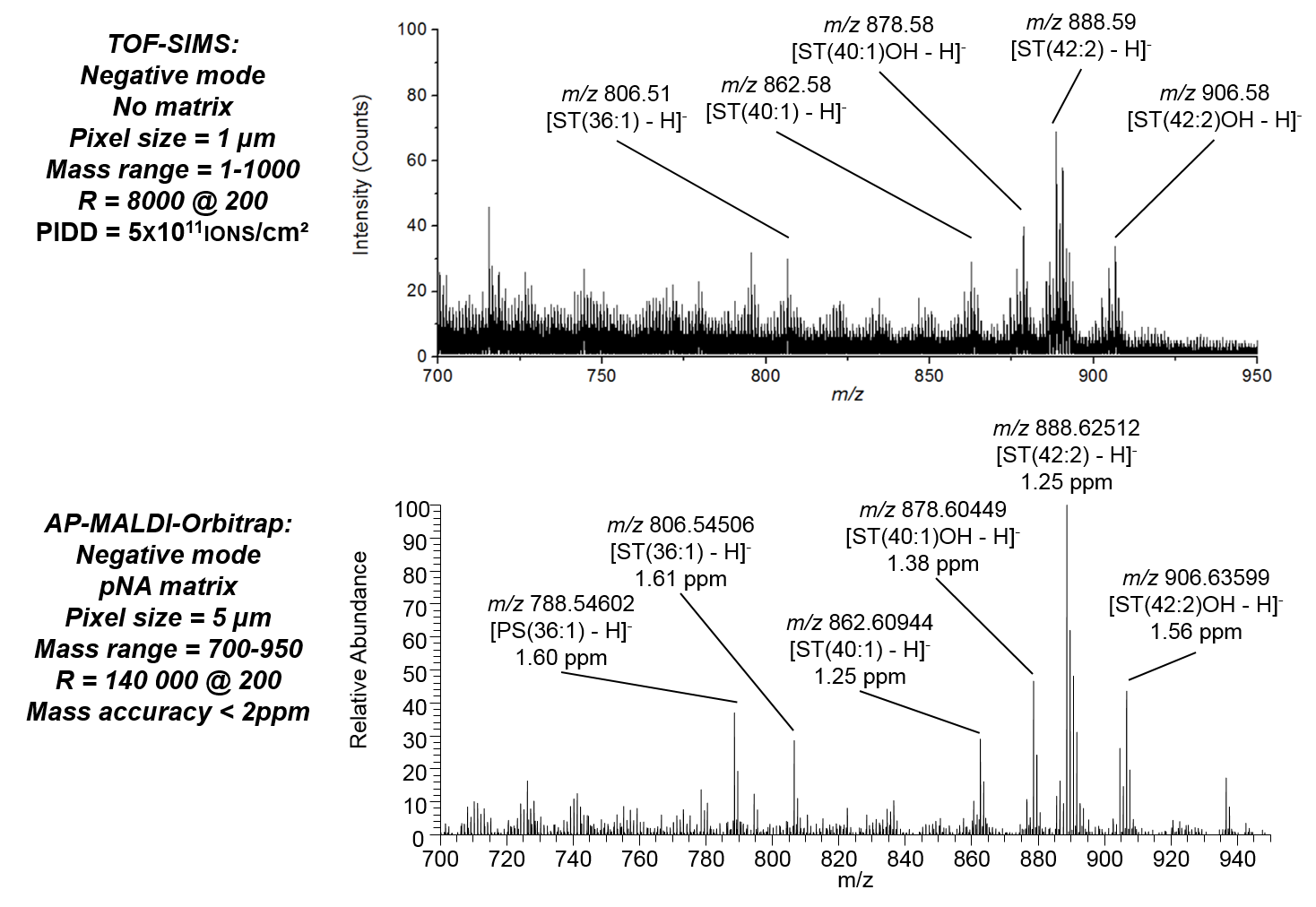


**Figure S3:** Mouse brain: Optical image of the hippocampus area corresponding to the region mapped by AP-MALDI-MS, and the black square corresponds to the region imaged by the TOF-SIMS (A); AP-MALDI-MS image in negative ion mode in the “gray” colormap: [ST(42:2) - H]^-^ at *m/z* 888.62512 (B); Adjacent juxtaposition of ten TOF-SIMS images in negative mode in the “gray” colormap: [ST(42:2) - H]^-^ at *m/z* 888.59 (C).


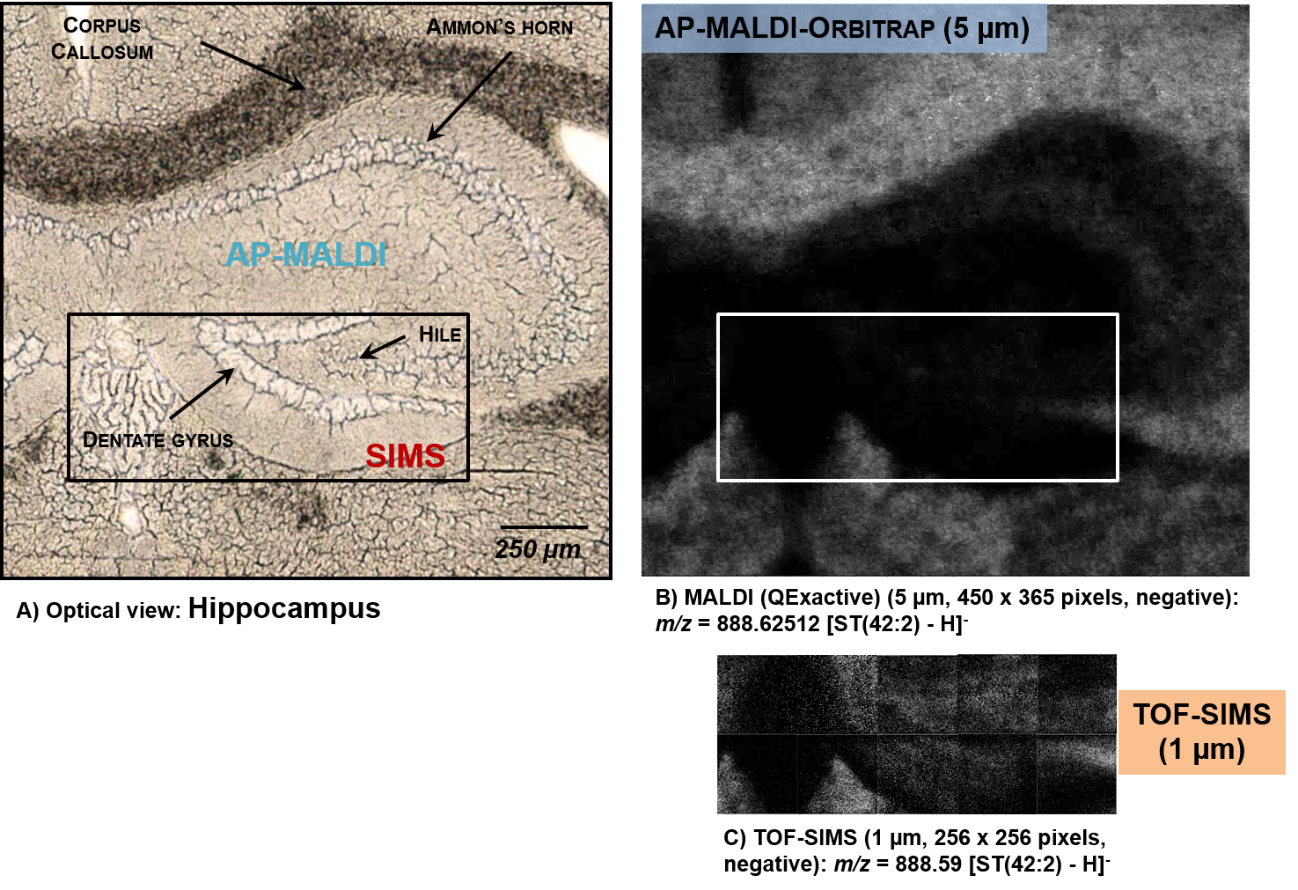

Supplement: Supplementary file 1 — Figure S1: TOF‐SIMS mass spectra in positive ion mode in the mass range of m/z 150 – 250 (A), and m/z 300 – 500 (B). Table S1: Product ions detected in MS2 spectra from SMALDI‐MS in positive ion mode. Figure S2: TOF‐SIMS mass spectrum in negative ion mode (top) and AP‐MALDI‐MS mass spectrum in negative ion mode in mass range of m/z 700 – 950 (bottom). The lipids identified are assigned with the experimental mass measured and root mean square (RMS) values were calculated for the AP‐MALDI acquisition. Figure S3: Mouse brain: Optical image of the hippocampus area corresponding to the region mapped by AP‐MALDI‐MS, and the black square corresponds to the region imaged by the TOF‐SIMS (A); AP‐MALDI‐MS image in negative ion mode in the “gray” colormap: [ST(42:2) ‐ H]‐ at m/z 888.62512 (B); Adjacent juxtaposition of ten TOF‐SIMS images in negative mode in the “gray” colormap: [ST(42:2) ‐ H]‐ at m/z 888.59 (C). [file RCM-32-159-s001.docx]
